# Economic burden of tuberculosis in Tanzania: a national survey of costs faced by tuberculosis-affected households

**DOI:** 10.1186/s12889-022-12987-3

**Published:** 2022-03-29

**Authors:** Andrew Martin Kilale, Andrea Pantoja, Bhavin Jani, Nyagosya Range, Bernard James Ngowi, Charles Makasi, Melkisedeck Majaha, Chacha Dionis Manga, Sylvia Haule, Amani Wilfred, Pudensiana Hilary, Vishnu Mahamba, Emmanuel Nkiligi, Wilbard Muhandiki, Emmanuel Matechi, Beatrice Mutayoba, Nobuyuki Nishkiori, Julia Ershova

**Affiliations:** 1 National Institute for Medical Research, Muhimbili Medical Research Centre, Dar es Salaam, Tanzania; 2 World Health Organization, Global TB Programme, Geneva, Switzerland; 3 World Health Organization, Country Office, Dar es Salaam, Tanzania; 4 University of Dar es Salaam, Mbeya College of Health Sciences, Mbeya, Tanzania; 5 KNCV Tuberculosis Foundation, Dar es Salaam, Tanzania; 6 Gender, Elderly and Children, National Tuberculosis and Leprosy Program, Ministry of Health, Community Development, Dodoma, Tanzania; 7 US Centers for Disease Control and Prevention, Atlanta, GA USA

**Keywords:** Catastrophic Cost, Tuberculosis, Cost drivers, Tanzania, National Survey, Out-of-pocket Expenditures

## Abstract

**Background:**

Although tuberculosis (TB) care is free in Tanzania, TB-associated costs may compromise access to services and treatment adherence resulting in poor outcomes and increased risk of transmission in the community. TB can impact economically patients and their households. We assessed the economic burden of TB on patients and their households in Tanzania and identified cost drivers to inform policies and programs for potential interventions to mitigate costs.

**Methods:**

We conducted a nationally representative cross-sectional survey using a standard methodology recommended by World Health Organization. TB patients of all ages and with all types of TB from 30 clusters across Tanzania were interviewed during July – September 2019. We used the human capital approach to assess the indirect costs and a threshold of 20% of the household annual expenditure to determine the proportion of TB-affected households experiencing catastrophic cost. We descriptively analyzed the cost data and fitted multivariable logistic regression models to identify potential predictors of catastrophic costs.

**Results:**

Of the 777 TB-affected households, 44.9% faced catastrophic costs due to TB. This proportion was higher (80.0%) among households of patients with multi-drug resistant TB (MDR-TB). Overall, cost was driven by income loss while accessing TB services (33.7%), nutritional supplements (32.6%), and medical costs (15.1%). Most income loss was associated with hospitalization and time for picking up TB drugs. Most TB patients (85.9%) reported worsening financial situations due to TB, and over fifty percent (53.0%) borrowed money or sold assets to finance TB treatment. In multivariable analysis, the factors associated with catastrophic costs included hospitalization (adjusted odds ratio [aOR] = 34.9; 95% confidence interval (CI):12.5–146.17), living in semi-urban (aOR = 1.6; 95% CI:1.0–2.5) or rural areas (aOR = 2.6; 95% CI:1.8–3.7), having MDR-TB (aOR = 3.4; 95% CI:1.2–10.9), and facility-based directly-observed treatment (DOT) (aOR = 7.2; 95% CI:2.4–26.6).

**Conclusion:**

We found that the cost of TB care is catastrophic for almost half of the TB-affected households in Tanzania; our findings support the results from other surveys recently conducted in sub-Saharan Africa. Collaborative efforts across health, employment and social welfare sectors are imperative to minimize household costs due to TB disease and improve access to care, patient adherence and outcomes.

**Supplementary Information:**

The online version contains supplementary material available at 10.1186/s12889-022-12987-3.

## Background

Tuberculosis (TB) is an infectious disease of global public health concern, especially in developing countries [1]. Globally, the disease contributes to high incidences of ill health causing high morbidity and mortality; an estimated 10.0 million people fell ill with TB in 2020. Despite the fact that TB is curable, it is a leading cause of death among individuals, causing 1.3 million deaths among HIV-negative people and 214,000 among HIV-positive people in 2020 [1].

While TB affects all income quintiles of the population, it disproportionately affects economically and socially disadvantaged persons, and therefore, reinforce barriers that keep them from seeking treatment [2–4]. Although TB patients in most high TB-burden countries have access to free anti-TB drugs, they often incur expenses on transport, nutrition or time costs while seeking care [5]. The TB-associated costs faced by patients may thwart access to diagnosis and care in the first place for those with constrained incomes, and, as these costs reduce family income, patients and their households are more vulnerable to social and financial hardship [6, 7]. Consequently, treatment adherence and patients’ outcomes are compromised, increasing the risk of transmission in the community, hindering efforts to address TB [7, 8]. Social and financial protection are a key requirement to successfully end TB within the agenda of Sustainable Development Goals [9]. One of the three targets for the WHO End TB Strategy is that no TB patient or their household should face catastrophic costs due to TB, defined as total costs borne by patients in tuberculosis treatment, exceeding a threshold of 20% of the household’s annual pre-TB income.

Tanzania is the largest and most populous East African country with an estimated population of 58 million in 2019 [10]. Between 2007 and 2019, Tanzania recorded sustained economic growth and a persistent decline in poverty [11]. However, about half of the population continues to live below the international poverty line of $1.90 per person per day (in 2011 purchasing power parity) and a significant proportion of the population remains vulnerable to falling into poverty. Tanzania is among the 30 WHO-identified high TB burden countries globally; in 2019, a total of 82,166 cases of all types of TB were notified [12]. TB services in Tanzania are provided through the primary health care network under the stewardship of the National TB Programme. TB treatment is organized in a patient-centered manner, where patients are treated mostly with a self-administered or home-based approaches. Directly observed therapy (DOT) at the health facility level is intended for fewer patients who requires close follow up and support. Hospitalization is considered only for patients with severe medical conditions. TB care is offered free in Tanzania to ensure universal access and to reduce the socioeconomic burden associated with the disease. However, to the best of our knowledge, programmatic data on costs incurred by patients while on TB care and the impact of these costs, including catastrophic costs, have not previously been collected in Tanzania.

We assessed the economic burden of TB and identified the main cost drivers among TB patients in Tanzania. Specifically, we aimed to determine the direct and indirect costs due to TB diagnosis and care (including during the health seeking period that led up to a TB diagnosis), estimate the proportion of households experiencing catastrophic costs due to TB, and identify potential predictors of catastrophic cost.

## Methods

### Study design

We conducted a cross-sectional survey of a nationally representative sample of TB patients who were diagnosed and treated under the national TB programme. The survey followed the WHO-recommended method to assess costs faced by TB patients and their households with the standard data collection instrument adapted to the national context [13]. The country specific adaptation included questions related to the health system structure, socio-economic positions (education, occupation and employment), the household assets and expenditures (Supplement 1). Clusters, each consisted of one or multiple health facilities, were selected based on probability proportional to the size of the number of TB patients notified in 2017. TB patients from 30 randomly selected clusters across the country were interviewed from July to September 2019.

The sample size was calculated for a desired precision for a proportion with the following standard formula.

$$N = DEFF\mathrm{ x}\begin{array}{c}{1.96}^{2}\mathrm{n}(1-{\uppi }_{\mathrm{g}}){\uppi }_{\mathrm{g}}\\ {d}^{2}(n-1)+{1.96}^{2}(1- {\pi }_{g}) {\pi }_{g}\end{array}$$

where: (1) the estimated percentage of TB affected households experiencing catastrophic costs due to TB illness (*π*_*g*_) is assumed to be 50% (as the most conservative estimate that gives the largest sample size); (2) assuming 5% absolute precision (*d*); (3) assuming a design effect (*DEFF*) of 2; and the total number of TB notifications (*n*) registered in 2017 was set as 65,908 for the finite number correction. Accordingly, a minimum sample size of 764 was needed. Considering the balance between logistical feasibility and statistical acceptability (e.g., minimizing sampling errors), a number of clusters was set as 30 with 26 patients to be enrolled in each cluster. All TB patients of any age and any type of TB were eligible for enrollment if they were registered for treatment, attending a health facility within a sampled cluster, received at least two weeks of intensive or continuation phase treatment, and consented to the study. Patients coming to health facilities for follow up visits were enrolled consecutively until the required number of patients in each facility was reached. Each patient was interviewed only once during the treatment period [13]. Parents or guardians answered the questions for children under 18 years old. MDR-TB in our survey represents a treatment category, where the patients were receiving second-line treatment according to the Tanzanian treatment guidelines. Data were collected electronically using ODK platform during the interview and stored in a web-based hosting server.

### Data collection

During the interview we collected information on TB-related costs incurred by respondents and their households, their clinical, demographic, socio-economic characteristics (e.g., income of patient and of household), as well as information on household assets and expenditures. TB-related costs included direct medical (consultation fees, medicines, diagnostic tests), direct non-medical (travel fees, accommodation, and food), and indirect (time lost while seeking and receiving care) costs. In addition, we collected data on the coping mechanisms, including selling assets and borrowing money, and social consequences such as job loss, disruption of schooling and social exclusion.

Data were collected for the treatment phase the patient was in at time of interview. Only patients in intensive phase, both DS-TB and MDR-TB, were also asked about costs incurred from the onset of symptoms until start of treatment (pre-diagnosis phase). To estimate the total costs incurred during the patient’s current treatment phase we extrapolated costs using the time left to complete the phase. To estimate patient costs for the entire TB episode (both phases of treatment), we imputed costs based on data from patients in other phases of treatment according to WHO methodology [13].

### Statistical analysis

Data analysis followed the standard analysis protocol developed by WHO [13]. Analysis was performed primarily with Stata v.16 SE (Stata Corp, College Station, TX), followed by data visualization and statistical modelling with R 4.0.2 (CRAN: Comprehensive R Archive Network at https://cran.r-project.org/). For descriptive statistics, we summarized binary variables by percentage and continuous variables by mean and median.

To estimate the costs from the other phase of treatment, we use the median reported costs from other patients that were sampled in that treatment phase. For example, a patient in the intensive phase during the interview would report the costs experienced so far in the treatment and diagnosis; in the analysis, the costs during the continuation phase for this patient would be the median costs of all the other patients in the continuation phase. In this calculation, costs from DS-TB and MDR-TB patients are considered separately. Results are presented for the total sample and disaggregated for drug-susceptible (DS-TB) and MDR-TB cases. Although the sample size was small for MDR-TB patients, we provide cost estimates for this subgroup as the drug resistant status has been identified as one of the important risk factors for catastrophic costs.(ref to the global TB report) Costs were converted to US Dollars (US$) using the average annual exchange rate during 2019 of US$ 1 = 2307.06 Tanzanian Shillings [14].

We used a human capital approach to value the indirect costs, meaning that time was valued with the hourly income reported by enrolled individuals. The Tanzanian team of this survey agreed that the human capital approach would produce more robust estimates in the country where the informal economy is large. Missing income data was imputed by a predicted value from a multiple liner regression model using household assets as independent variables and income as the dependent variable [13].

We measured the proportion of households experiencing catastrophic costs due to TB among enrolled individuals. Catastrophic costs were defined as TB-related costs (direct and indirect) exceeding 20% of the annual household expenditure. We used 20% as the threshold to be in line with the WHO indicator and methodology [13]. To identify risk factors for catastrophic costs, we conducted multivariate logistic regression analysis using the occurrence of catastrophic costs as a binary outcome variable. Age group, sex, drug-susceptibility, treatment history, recorded HIV status, income quintile, hospitalization, treatment modality, insurance status, urban or rural settings are included as explanatory variables. No interaction term was used. A crude odds ratio was estimated for each of the explanatory variables by a univariable logistic regression model. To determine the best model, the stepwise backward approach was employed. The Akaike Information Criterion (AIC) was used to compare models in each step until we reach the best model that produces the minimum AIC [15]. We presented adjusted odds ratios for the variables retained in the final model.

We also assessed the increase in the households’ poverty resulting from incurring in costs for TB care using the US$ 1.90/day international poverty line of The World Bank [16].

As a sensitivity analysis, we varied the 20% threshold for catastrophic costs to see how different thresholds would affect the proportion of households experiencing catastrophic costs [17–20]. In order to compare the final result, we also measured the proportion of households experiencing the catastrophic costs based on the output approach; this means we measured indirect costs using the household income before and after TB (income loss) during TB episode.

### Ethical issues

The study was approved by the National Health Research Ethics Committee of Tanzania (FWA00002632, expires: 9/22/2021) and the U.S. Centers for Disease Control and Prevention (CDC), Center for Global Health (CGH) (Project number: 2018–277). The study was reviewed in accordance with the U.S. CDC human research protection procedures and determined to be research, but CDC investigators did not interact with human subjects or have access to identifiable data or specimens for research purposes. Written informed consent or assent was obtained from all participants.

## Results

Out of the 784 individuals that were invited to participate in the study, 777 (99.1%) were eligible and participated. On average, participants were 39 years old (range 1–91); 37.2% were females. Three quarters (75.1%) of the participants had completed primary education. The employment rate of people in this survey diminishes after diagnosis of TB: 82% were employed before contracting TB, but only 36.4% were employed at the time of the interview (Table 1). Sixty-one percent of respondents lived in an urban or semi-urban setting. Overall, 25 (3.2%) patients were being treated for MDR-TB at the time of the survey, and about a third of these had already been treated for TB in the past (Table 1). Thirty-nine per cent (39.0%) of sampled patients were in the intensive phase and 12% of sampled patients reported more than 4 weeks between experiencing symptoms and diagnosis. More than half of the enrolled TB patients (55.6%) were on self-administered TB treatment. The median household income per year was reported as US$ 1809 before contracting the disease, and reduced to US$ 1548 per year during the TB episode (Table 2).

**Table 1:** Socio-demographic and economic characteristics of study population (*n* = 777)

Characteristics	DS-TB	MDR-TB	All
	***n* = 752**	***n* = 25**	***n* = 777**
**Gender, *n (%)***			
Female	285 (37.9)	4 (16.0)	289 (37.2)
Male	467 (62.1)	21 (84.0)	488 (62.8)
**Age in years, mean (SD)**	39.5 (18.9)	42.5 (14.9)	39.6 (18.8)
**Treatment phase, *n (%)***			
Intensive	292 (38.8)	11 (44.0)	303 (39.0)
Continuation	460 (61.2)	14 (56.0)	474 (61.0)
**Treatment history, *n (%)***			
New	677 (90.0)	17 (68.0)	694 (89.3)
Relapse/Retreatment	75 (10.0)	8 (32.0)	83 (10.7)
**Treatment modality, *n (%)***			
Self-administration	420 (55.8)	12 (48.0)	432 (55.6)
Home-based DOT	317 (42.2)	3 (12.0)	320 (41.2)
Facility-based DOT	15 (2.0)	10 (40.0)	25 (3.2)
**Treatment delay, *n (%)***			
> 4 weeks since start of symptoms	86 (11.4)	11 (44.0)	94 (12.3)
**Patient's education status, *n (%)***	591	20	611
No education	4 (0.7)	0	4 (0.7)
Primary school	444 (75.1)	15 (75.0)	459 (75.1)
Secondary/high school	121 (20.5)	5 (25.0)	126 (20.6)
University and higher	22 (3.7)	0	22 (3.6)
**Employed before TB, *n (%)***	622 (82.7)	22 (88.0)	644 (82.9)
**Employed after TB, *n (%)***	278 (37.0)	5 (20.0)	283 (36.4)

*Abbreviations*: *DS-TB* Drug-susceptible TB, *MDR-TB* Multi-drug resistant TB, *DOT* Directly Observed Therapy, *SD* Standard deviation, *IQR* Interquartile range

**Table 2:** Income characteristics of study population (*n* = 777), annual income

	**DS-TB (*n* = 752)**		**DR-TB (*n* = 25)**		**All (*N* = 777)**	
	**Mean (SD)**	**Median (IQR)**	**Mean (SD)**	**Median (IQR)**	**Mean (SD)**	**Median (IQR)**
**BEFORE TB: Annual household income, US$**	2351 (2,211)	1809 (1,040 – 2,932)	2907 (2,974)	2190 (1,560–3,121)	2369 (2,239)	1809 (1,040–2,976)
**DURING TB: Annual household income, US$**	1828 (1,808)	1548 (768–2,341)	1646 (807)	1548 (1,219–2,190)	1822 (1,785)	1548 (768–2,341)

### Cost and drivers of TB care

The total median cost incurred by TB patients during their illness was US$ 154.9 (interquartile range (IQR): 96.7–257.6) (Table 3 Total Cost). The median cost among the MDR-TB patients in our sample was US$ 312.0 (IQR: 212.2–604.9) and among DS-TB patients it was US$ 150.6 (IQR: 95.2–247.8). The highest costs during a TB episode were incurred during treatment (91.1% of the total costs), compared to costs during the pre-diagnosis period.

**Table 3:** Estimated total costs of TB care during a TB episode faced by patients, study population (*n* = 777)

Cost category (777 TB patients)	Mean (SD) (US Dollars)	Median (IQR) (US Dollars)
**Pre-diagnosis**		
Medical	10.3 (15.9)	7.2 (7.2–7.2)
Non-medical	4.2 (6.8)	3.0 (3.0–3.0)
Indirect costs	8.9 (44.6)	3.1 (1.6–5.6)
**Post-diagnosis**		
Medical	29.3 (144.4)	0.0 (0.0–4.2)
Non-medical		
Travel	30.1 (37.1)	19.8 (9.2–39.1)
Accommodation	0.7 (3.3)	0.0 (0.0–0.0)
Food	13.8 (36.5)	5.0 (0.0–14.3)
Nutrition supplement	85.8 (91.7)	72.8 (0.0–121.4)
Indirect costs	79.7 (635.8)	16.8 (6.3–38.2)
**Overall**		
Medical	39.7 (145.3)	7.2 (7.2–23.2)
Non-medical	134.6 (120.6)	107.7 (66.5–175.0)
Indirect costs	88.6 (640.9)	20.4 (9.2–46.0)
**Total cost, all patients**	262.9 (764.3)	154.9 (96.7–257.6)
Total cost for DS-TB patients	252.0 (765.0)	150.6 (95.2–247.8)
Total cost for MDR-TB patients	592.5 (676.1)	312.0 (212.2–604.9)

*Abbreviations*: *DS-TB* Drug-susceptible TB, *MDR-TB* Multi-drug resistant TB, *SD* Standard Deviation, *IQR* Interquartile range

Costs borne by patients during pre-diagnosis period (before TB treatment) were driven by direct medical costs (44.0%), indirect costs (38.0%) and non-medical costs (18.0%). The costs during TB treatment were largely driven by non-medical costs, notably nutrition supplements (35.9%) and travel costs for drug pickup (12.6%). Indirect costs comprised one third (33.3%) of the total treatment costs, which was largely driven by the time lost during hospitalization and turnaround time for picking up drugs. The highest direct medical costs (12.2% of treatment costs) were incurred during follow-up visits, mainly for radiography/imaging tests and TB medicine (for both DS- and MDR-TB patients). Specifically, DS-TB patients paid most for x-rays (30.7% of direct medical costs faced by DS-TB patients), while MDR-TB patients paid most for other procedures (36.1% of direct medical costs faced by MDR-TB patients).

### Coping mechanisms and loss of productivity

**Fig. 1:**
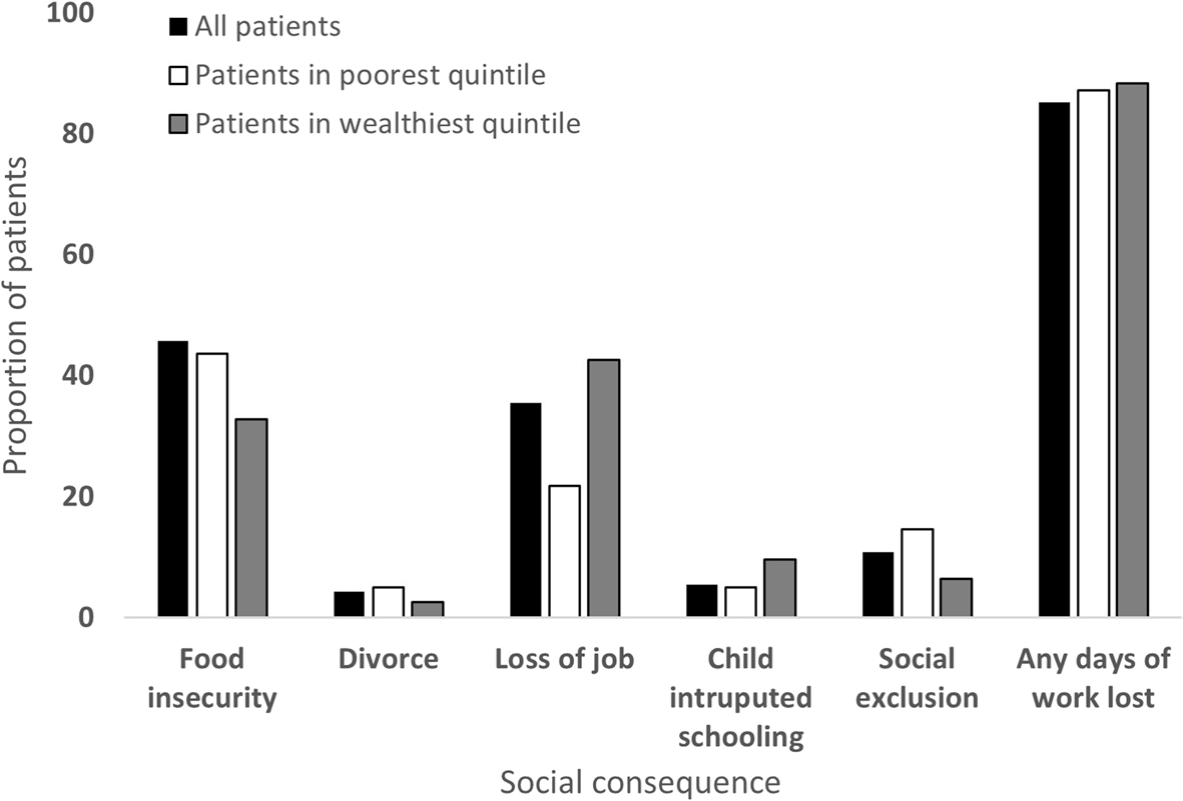
Social consequences of having TB as perceived by patients in the poorest/wealthiest expenditure quintile

Over half (53.0%) of the enrolled patients were unable to pay for TB treatment from their existing income alone or using a health insurance, and they had to rely on borrowing or selling assets to finance costs associated with TB care. Only ninety-four participants (12.1% of the sample) had a health insurance. Four patients out of 5 (85.2%) lost at least one day of work due to TB diagnosis and treatment; this proportion was higher among MDR-TB patients (96.0%) compared to DS-TB patients (84.8%). One third of patients (35.5%) reported job loss because of TB; wealthier percentiles (42.6%) were more affected than poorer percentiles (21.8%) (Fig. 1). A higher proportion of patients from the poorer percentiles suffer from social exclusion due to TB compared to patients in wealthier percentiles.

### Catastrophic costs experienced by TB-affected households

**Fig. 2:**
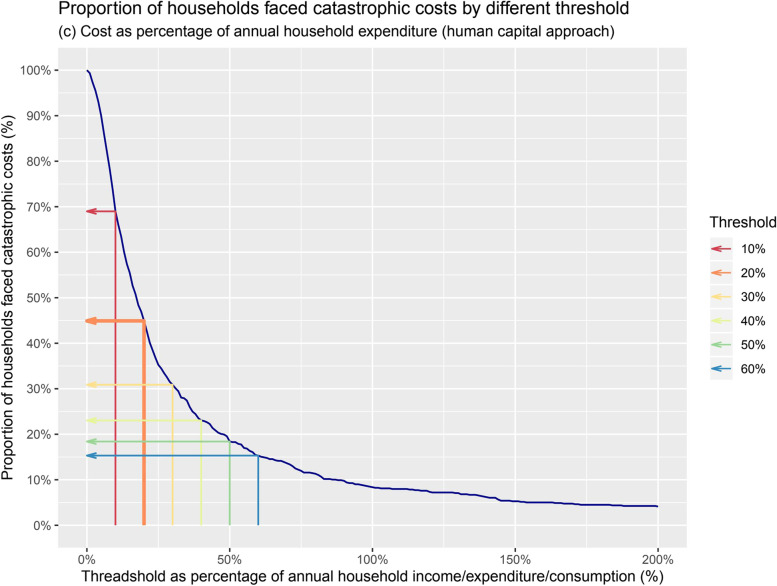
Proportion of households with TB patients facing catastrophic costs, by different thresholds, 777 TB-affected households

The proportion of households experiencing catastrophic costs at 20% threshold of annual household expenditure was 44.9% (349/777) (95% confidence interval (CI): 41.3–48.4) (Table 4, Fig. 2). This ratio was estimated at 80.0% (95%CI: 59.2–93.1) for the sample of MDR-TB patients; and at 43.7% (95%CI: 40.1–47.3) for DS-TB patients. In our sample, 7.7% of patients were hospitalized, 13.8% were living in semi-urban areas, 38.5% in rural areas, 3.2% were MDR patients, and 3.2% received treatment at the facility. Independent predictors of catastrophic costs included hospitalization (adjusted odds ratio [aOR] = 35.0; 95%CI: 12.5–146.1), living in semi-urban (aOR = 1.6; 95%CI: 1.0–2.5) or rural areas (aOR = 2.6; 95%CI: 1.8–3.7), having MDR-TB (aOR = 3.4; 95%CI: 1.2–10.9), and facility-based directly-observed treatment (aOR = 7.2; 95%CI: 2.4–26.6) (Table 5).

**Table 4:** Proportion of households experiencing catastrophic costs due to TB, by different thresholds ( *n* = 777)

Threshold as % of annual household expenditure	Households facing catastrophic costs n (%)
(The End TB Strategy indicator)	
10%	536 (69.0)
**20%**	**349 (44.9)**
30%	240 (30.9)
40%	179 (23)
50%	143 (18.4)
60%	119 (15.3)

**Table 5:** Risk factors for experiencing catastrophic costs due to TB among 777 TB-affected households in Tanzania

Variable	Total *n* = 777	Number and proportion of incurred catastrophic cost n (%)	*P*-value^a^	OR univariable (95%CI)	aOR^b^ multilevel (95%CI)
**Age group**					
0–14	73	34 (46.6)	0.223	Reference	
15–24	86	30 (34.9)		0.61 (0.32–1.16)	
25–34	154	63 (40.9)		0.79 (0.45–1.39)	
35–44	164	73 (44.5)		0.92 (0.53–1.60)	
45–54	139	65 (46.8)		1.01 (0.57–1.78)	
55–64	82	43 (52.4)		1.26 (0.67–2.39)	
> 65	79	41 (51.9)		1.24 (0.65–2.35)	
**Sex**					
Female	289	126 (43.6)	0.62	Reference	
Male	488	223 (45.7)		1.09 (0.81–1.46)	
**Drug susceptibility status**^**c**^					
DS-TB	752	329 (43.8)	0.001	Reference	Reference
MDR-TB	25	20 (80.0)		5.14 (2.06–15.58)	3.36 (1.20–10.92)
**Treatment history**					
New	694	314 (45.2)	0.67	Reference	
Retreatment	83	35 (42.2)		0.88 (0.55–1.39)	
**Recorded HIV status**^**c**^					
Negative	528	237 (44.9)	0.923	Reference	
Positive	234	106 (45.3)		1.02 (0.75–1.38)	
Unknown	15	6 (40.0)		0.82 (0.27–2.30)	
**Income quintile**					
Wealthiest	155	69 (44.5)	0.64	Reference	
Fourth	154	66 (42.9)		0.93 (0.60–1.47)	
Third	108	43 (39.8)		0.82 (0.50–1.36)	
Second	192	93 (48.4)		1.17 (0.77–1.79)	
Poorest	168	78 (46.4)		1.08 (0.70–1.68)	
**Hospitalizations during current phase**					
No	717	292 (40.7)	< 0.001	Reference	Reference
Yes	60	57 (95.0)		27.65 (10.10–114.13)	34.97 (12.50–146.11)
**Treatment modality**					
Self-administration	432	195 (45.1)	< 0.001	Reference	Reference
Home-based DOT	320	133 (41.6)		0.86 (0.64–1.16)	0.90 (0.63–1.27)
Facility-based DOT	25	21 (84.0)		6.38 (2.38–22.15)	7.19 (2.42–26.56)
**Insurance**					
No	683	311 (45.5)	0.41	Reference	
Yes	94	38 (40.4)		0.81 (0.52–1.25)	
**Delay before diagnosis**					
No delay	683	301 (44.1)	0.24	Reference	
Delayed	94	48 (51.1)		1.32 (0.86–2.04)	
**Settings**^**d**^					
Urban	371	136 (36.7)	< 0.001	Reference	Reference
Semi-urban	107	44 (41.1)		1.21 (0.77–1.87)	1.58 (0.98–2.53)
Rural	299	169 (56.5)		2.25 (1.65–3.07)	2.57 (1.82–3.66)

*Abbreviations*: *CI* Confidence interval, *HIV* Human immunodeficiency virus, *OR* Odds ratio, *Aor* Adjusted OR, *DS-TB* Drug-susceptible TB, *MDR-TB* Multi-drug resistant TB, *DOT* Directly observed therapy

^a^Chi-square test

^b^Based on the final logistic regression model selected with stepwise backward selection which was guided by Akaike’s information criterion (AIC)

^c^MDR TB and HIV status of patients were retrieved from patients’ charts

^d^Definitions used in Tanzania for each setting:

Urban: city of a region

Semi-urban: township of a district

Rural: village other than the township of district

Poverty increased after a TB episode from 78.5% of households below the poverty line before TB to 85.0% below the poverty line at the time of the survey. The sensitivity analysis shows that as the catastrophic cost threshold increases, the proportion of households facing catastrophic costs decreases accordingly: at a 40% threshold, 23.0% of households still face catastrophic costs due to TB (Table 4). Use of the output approach for calculation of indirect costs resulted in 52.6% of households facing catastrophic costs at the same 20% threshold.

## Discussion

We conducted the first nationwide survey to assess the costs and the financial hardship faced by TB patients and their households in Tanzania. We found that almost half of TB-affected households in our sample experienced catastrophic costs due to TB diagnosis and treatment. Our data showed that the proportion of households facing catastrophic costs was higher for MDR-TB patients compared to DS-TB patients; albeit our small sample of MDR-TB patients in Tanzania. The median total costs due to TB diagnosis and treatment was US$ 155 per patient. In our sample, patients treated from MDR-TB faced on average two times higher costs compared to patients with DS-TB. Costs during TB treatment were higher than costs during the pre-diagnosis period and were driven mainly by nutritional supplements and indirect costs.

As of September 2020, 19 countries have completed TB patient cost surveys using the recommended WHO methodology [12] Among countries in Africa, the proportion of patients experiencing catastrophic costs in Tanzania (44.9%) were similar to estimates in Nigeria (51.4%) and Uganda (53.1%), higher than the estimates from Kenya (26.5%), and lower than estimates from Ghana (64.1%) and Zimbabwe (77.0%) [20–23]. Nutritional supplements and indirect costs were the highest expenses for TB patients in Tanzania, as well as in Ghana, Kenya, and Uganda. Nigeria found that the largest cost drivers were indirect costs and additional food bought while accessing TB services. All studies found that costs for DR-TB patients could be more than double of those for DS-TB patients, mainly because of direct non-medical costs (particularly nutrition supplements) and indirect costs. Although our results for MDR-TB patient are not representative, they are consistent with findings from elsewhere in sub-Saharan Africa. Additional assessments focusing on MDR-TB patients may be needed to obtain representative cost estimates for this population.

Our results demonstrated that for the most DS-TB patients the highest financial burden during the TB episode was due to the indirect costs, i.e., due to loss of work time evaluated using a human capital approach. Indirect costs for DS-TB patients were mainly driven by hospitalization and the frequency and travel time to pick up drugs. Decentralizing the service delivery points for dispensing TB medicines may improve access to the services and reduce the time spent by patients. We have found that accessing the Directly Observed Therapy (DOT) centers represent the highest indirect costs during their treatment for MDR-TB patients. This could be reduced by expanding oral regimens through community-based treatment support as recommended by WHO [22].

All African national TB patient cost surveys published to date revealed that costs for nutrition supplements are one of the highest out-of-pocket expenditures for TB patients. In Tanzania, our data suggest that the total costs during TB treatment were driven by costs associated with nutritional supplements, especially among MDR-TB patients. Certainly, it will be useful to take a closer look at what type of nutrition supplements were used by TB patients. A recent systematic review indicated that while nutritional supplements may increase the weight of the patients they may not necessarily be the main factor to affect positive TB treatment outcomes [23]. It may be useful for patients if the food support program of the country is extended to cover all TB patients with moderate to severe malnutrition and malnourished children in TB households.

Despite the goal of providing TB treatment ‘free of charge’ in public sector in Tanzania, some direct medical costs for treatment were reported by TB patients in our study—representing about a tenth of the total expenditures. National patient cost surveys in other countries also revealed direct medical cost experienced by patients during TB treatment. In Tanzania medical costs emerged mainly during follow-up visits for DS-TB and MDR-TB patients. Specifically, DS-TB patients paid most for radiographies, while MDR-TB patients paid most for other diagnostic procedures. During the pre-diagnosis phase, medical costs are almost half of costs before diagnosis. Minimizing expenses for medical services during TB treatment is likely to reduce costs for patients and mitigate financial burden for their households. Reinforcing measures such as active-case finding would contribute to reduce costs faced by patients.

The significant results and conclusions of this survey have informed policy makers in Tanzania. An early impact is that the government has agreed to include TB in the list of partial disabilities which warrants social protection for TB patients.

### Survey limitations

Our survey had several limitations. We used a cross-sectional design with forward extrapolations on patient costs. While this is a practical method given the context of the study and is recommended by WHO, the estimated costs may not reflect the true costs experienced by the patients due to the illness. Longitudinal methods of determining patient costs may provide more accurate measures, although are more complex to implement. However, we also attempted to analyze costs as reported at the time of interview, allowing to have conclusions about the cost per TB service in each phase of treatment. In addition, patients might not remember accurately the amounts spent in seeking care and during treatment (recall bias). We attempted to minimize this by asking patients only about the treatment phase they were in; furthermore, we used prompts to assist patients in recalling costs. We presented disaggregated results for the MDR-TB group although this sample is not nationally representative. Nevertheless, we believe that MDR-TB patients experience large burden, therefore the information collected and analysed needs to be shared for comparability with results from elsewhere and to inform future assessments focusing on MDR-TB patients. Finally, we did not interview patients who were “lost to follow up” – some of these patients might have disrupted treatment due to financial difficulty and therefore we may have underestimated the proportion of patients with catastrophic costs. We also did not interview the relatives of patients who had died due to TB because of our finite resources and the complexities of conducting such survey in Tanzania. We acknowledge that this is an important area for future research, also recognized by the WHO, that designed these surveys.

## Conclusion

For the first time in Tanzania, we have provided national cost estimates and identified a range of potential drivers of costs and of catastrophic cost, which can inform programmatic and policy decisions, and enable effective translation of the findings into actions. Similar to other countries in sub-Saharan Africa, we found that the economic burden for households due to TB in Tanzania is high. Simplified access to TB services, enhanced access for TB patients to national nutrition programs, assurance of the free provision of TB medicines and diagnostic procedures, and partnership with social welfare sectors could ameliorate the negative consequences of TB, experienced by TB patients and their families in Tanzania.

## Supplementary Information

**Additional file 1.**

## Data Availability

The datasets generated and/or analysed during the current study are not publicly available due to lack of resources required to prepare the dataset for public but can be available from the corresponding authors on reasonable request.
