# Supplementary material for: Economic burden of tuberculosis in Tanzania: a national survey of costs faced by tuberculosis-affected households
Source: BMC Public Health. 2022 Mar 29;22:600. doi: 10.1186/s12889-022-12987-3 (PMC8961947; doi:10.1186/s12889-022-12987-3)
Supplement: Supplementary file 1 — Additional file 1. [file 12889_2022_12987_MOESM1_ESM.docx]

**Ministry of Health, Community Development, Gender, Elderly and Children**

**National Tuberculosis and Leprosy Program**

**
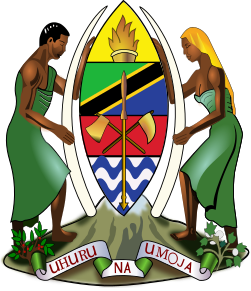
**

***Assessment of the economic burden***

***incurred by tuberculosis patients and their households on diagnosis and treatment of tuberculosis in Tanzania in 2019***

***(Tanzania Patient Cost Survey)***

**Survey Questionnaire**

*National Tuberculosis and Leprosy Program*

*Department of Preventive Services*

*Ministry of Health, Community Development, Gender, Elderly and Children*

*University of Dodoma, Faculty of Arts and Community Development*

*Building Number 11*

*P. O. Box 743, 40478* ***Dodoma***

E-mail : [info@ntlp.go.tz](mailto:info@ntlp.go.tz)

**Supplement Material 1:** Questionnaire for Tanzania TB Patient Cost Survey 2019

**
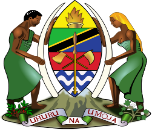
**

***Part I. Informed consent***

**At this point, the interviewer should provide the patient with the information sheet, explain the study and obtain written informed consent.**

**Patient PCS ID _____________________ TB Registration No _________________________**

***Part II. Patient information to be obtained from TB treatment card before interview***

| **Question** | **Answer categories** *(circle appropriate number or fill answer on the answer line)* | ***Action for interviewer***  *The questions in Part II are not part of the interview and should be pre-filled before the interview* |
| --- | --- | --- |
| 1. Date of Interview | (Day/month/year) ……/……/……… | \| *The region and district are the locations where the patient is being interviewed*  *The name of the facility where the patient is being interviewed* \| \| --- \| |
| 1. Name of Region |  |  |
| 1. Name of District |  |  |
| 1. Place of interview (facility name) |  |  |
| 1. Interviewer Name |  |  |
| 1. Category of treating facility | A: Public Health Facility   1. Dispensary 2. Health center 3. District Hospital 4. Regional Referral Hospital 5. Zonal referral Hospital 6. National Referral Hospital   B: NGO/charitable/private Facility   1. Dispensary 2. Health center 3. District Hospital 4. Regional referral Hospital 5. Zonal referral Hospital 6. Other____________________________ | *The "treating facility" is the place where the patient's treatment card is kept* |
| 1. Sex | 1. Male 2. Female |  |
| 1. Age of patient: | ____years |  |
| 1. Date of first bacteriological TB test | (Day/month/year) ……/……/………  not done or unknown |  |
| 1. Place of diagnosis | A: Public Health Facility   1. Dispensary 2. Health center 3. District Hospital 4. Regional Referral Hospital 5. Zonal referral Hospital 6. National Referral Hospital   B: NGO/charitable/Private Facility   1. Dispensary 2. Health center 3. District Hospital 4. Regional Referral Hospital 5. Zonal referral Hospital 6. Other____________________________ |  |
| 1. Type of TB | 1. Pulmonary, bacteriologically confirmed  2. Pulmonary, bacteriologically unconfirmed  3. Extra-pulmonary |  |
| 1. On MDR-TB treatment | 1. Yes 2. No |  |
| 1. Total duration of planned treatment from start | ____________months intensive  ____________months continuation |  |
| 1. Treatment registration group | A: Drug susceptible TB  1. 1^st^ line, new  2. 1^st^ line, relapse  3. 1^st^ line, re-treatment after loss to follow-up  4. 1^st^ line, re-treatment after failure  B: MDR TB   1. MDR, new (initial MDR) 2. MDR, relapse 3. MDR, re-treatment after loss to follow-up 4. MDR, re-treatment after failure of first treatment with 1st-line drugs 5. MDR, re-treatment after failure of retreatment regimen with 1st-line drugs 6. Other, specify: ……………………… |  |
| 1. Start date of current TB treatment | (Day/month/year) ……/……/……… |  |
| 1. Is the patient currently in intensive or continuation treatment phase? | 1. Intensive phase, ___days of phase completed  2. Continuation phase, ___days of phase completed | *If patient has completed less than 2 weeks of the current treatment phase, exclude, or postpone interview. Interview takes place after a minimum 2 weeks have been completed.*  *Intensive phase for MDR-TB regimens is the initial treatment period which includes an injectable drug (usually 4 to 8 months).* |
| 1. HIV status   (as indicated on treatment card) | 1. Positive  2. Negative  3. Not tested  4. Unknown |  |
| 1. Weight of patient | _____ kg | *Weight can be recorded from the patient treatment card* |
| 1. Height of patient | _____ cm | *Height can be recorded from the patient treatment card* |

| ***Part III- Costs before the current TB treatment (to be filled for cases in intensive phase only)***  *For case interviewed in the continuation phase: skip to Part IV* | | |
| --- | --- | --- |
| **O*ut-of-pocket expenditure, reimbursements and time loss before and during TB diagnosis (before start of TB treatment)*** | | |
| **Question** | **Answer categories** *(check all that apply or fill answer on the answer line)* | ***Instructions and actions for interviewer*** |
| 1. For this episode of TB, when did you first experience symptoms of TB of this TB episode? | Weeks before treatment started:________ | *First construct a timeline of events, either starting with the first TB symptom, or start with time of TB diagnosis and work backwards. Use the locally adapted calendar with main seasonal events that the patient can relate to and use as a reference point for timing. To help the patient remember when the illness started, you can ask which TB symptom was first experienced, after having probed for cough, weight loss, chest pain, night sweats. If there is a problem defining the difference between TB symptoms and other health problems, ask which symptom led the patient to seek care, then ask when that symptom first occurred or became worse and started to worry the patient.* |
| 1. How much money and time did you spend for each of these visits before you were diagnosed with TB, including the visit when you actually received your diagnosis? | - See table below, and ask for each item - Fill one line per visit - *Enter in chronological order, using one of these provider categories for each visit, and entering how many weeks before TB treatment start each visit was.*   A: Public Health Facility   1. Dispensary 2. Health centre 3. District Hospital 4. Regional referral Hospital 5. Zonal referral Hospital 6. National referral Hospital   B: NGO/charitable/private Facility   1. Dispensary 2. Health centre 3. District Hospital 4. Regional referral Hospital 5. Zonal referral Hospital  - For all that don’t apply, mark/select NA - If there were payments for an item, but the patient cannot remember the amount, mark NR - Add more rows if more visits were made before diagnosis of TB!   Explanation of table headings:  Visits: Includes outpatient visits as well as hospitalizations. Should be filled in chronological order, 1st visit=visit 1.  Type of provider: fill in provider type according to categories in question 50 where patient sought treatment or advice.  Travel time: Hours or days spent to travel to and from facility  Time spent for visit: Fill in hours for outpatient visits and days for hospitalizations  Day charge: Fees for hospital days. Only for hospitalizations, and only to be filled if not covered by the cost items below (consultation fee, radiography etc.)  Consultation fee: Other charges, not covered under day charge, including direct payment to health care staff  Radiography and other imaging: out-of-pocket payments for imaging investigation (x-rays, CT-scan, ultrasound), TB-specific and other  Lab test fees: out-of-pocket payments for all tests, TB specific and others  Other procedures: out-of-pocket payments for biopsy, bronchial lavage etc. but not surgery unrelated to TB  Medicine fees: Any medicine (TB or other) prescribed before TB was diagnosed under NTP  Other, including nutritional supplements: any other treatments, such as nutritional supplements medically indicated  Travel: out-of-pocket payments for travel to the facility (does not include income loss), for both patient and any household member.  Food: out-of-pocket payments for additional food bought in relation to travelling the health care visit, and during visit or hospitalization, for both patient and any household member  Other, including accommodation: includes out-of-pocket payments related to renting a room/bed during health care visits, and any other non-medical payments related to health care visit, for both patient and any household member  **Health insurance reimbursement**: amount reimbursed to patient through medical insurance (private or social security) so far, does not include expected future reimbursement  **Out-of-pocket payments (gross):** Direct payment made to health-care providers by individuals at the time of service use, i.e. excluding prepayment for health services – for example in the form of taxes or specific insurance premiums or contributions. It is calculated as the sum of direct medical (A) and direct non-medical (B) costs. If patient cannot remember the details of costs above, ask for the total out-of-pocket payments of the visit, hospitalization.  Out-of-pocket payment (net): medical and non-medical out-of-pocket payments minus reimbursements. These net payments: should be calculated by supervisor after the interview. Not to be calculated during the interview. | |

|  |  |  |  | | **Medical out-of-pocket payments,**  **(Total TZS per visit)**  **(A)** | | | | | | | | **Non-medical out-of-pocket payments,**  **(Total TZS per visit)**  **(B)** | | | | **Out-of-pocket payments (A+B)**  **(Gross)** | **(C)** | **Out-of-pocket payments per stay**  **(A+B-C)**  **(Net)** |
| --- | --- | --- | --- | --- | --- | --- | --- | --- | --- | --- | --- | --- | --- | --- | --- | --- | --- | --- | --- |
| Visit | Weeks before treatment started | Type of provider | Travel time (hours) | Time spent for visit  (hours) | Day charges (for hospitalizations only)  A1 | Consultation fee  A2 | Radiography and other imaging  A3 | Lab  tests  A4 | Other procedures  A5 | Medicines  A6 | Other  incl. nutritional supplements  A7 | Medical payments total  ΣA1-7 | Travel  B1 | Food during health care visit or hospital stay  B2 | Other, including accommodation  B3 | Non-medical out-of-pocket payments (Total)  ΣB1-3 | Total out-of-pocket payments  (ΣA1-7) +  (ΣB1-3) | Health insurance reimbursement |  |
| 1^st^ |  |  |  |  |  |  |  |  |  |  |  |  |  |  |  |  |  |  |  |
| 2^nd^ |  |  |  |  |  |  |  |  |  |  |  |  |  |  |  |  |  |  |  |
| 3^rd^ |  |  |  |  |  |  |  |  |  |  |  |  |  |  |  |  |  |  |  |
| 4^th^ |  |  |  |  |  |  |  |  |  |  |  |  |  |  |  |  |  |  |  |
| 5^th^ |  |  |  |  |  |  |  |  |  |  |  |  |  |  |  |  |  |  |  |
| 6^th^ |  |  |  |  |  |  |  |  |  |  |  |  |  |  |  |  |  |  |  |
| 7^th^ |  |  |  |  |  |  |  |  |  |  |  |  |  |  |  |  |  |  |  |
| 8^th^ |  |  |  |  |  |  |  |  |  |  |  |  |  |  |  |  |  |  |  |
| 9^th^ |  |  |  |  |  |  |  |  |  |  |  |  |  |  |  |  |  |  |  |
| 10^th^ |  |  |  |  |  |  |  |  |  |  |  |  |  |  |  |  |  |  |  |

| ***Part IV. Cost during current TB/DR-TB treatment (to be filled for all patients)***  *Unless specified, this section refers to the patient’s current treatment phase only* | | | | | | | | | | | | | | | | | | | | | | | | | | | |  |
| --- | --- | --- | --- | --- | --- | --- | --- | --- | --- | --- | --- | --- | --- | --- | --- | --- | --- | --- | --- | --- | --- | --- | --- | --- | --- | --- | --- | --- |
| **Question** | | | | | | | **Answer categories** *(check all that apply or fill answer on the answer line)* | | | | | | | ***Instructions and actions for interviewer*** | | | | | | | | | | | | | |  |
| 1. Are you currently hospitalized? | | | | | | | 1. Yes 2. No | | | | | | | If yes, the cost data collected applies to the first row of the table question 20 | | | | | | | | | | | | | |  |
| 1. Have you been previously hospitalized during your current TB treatment phase and because of TB? If yes, how many times? | | | | | | | 1. Yes Times _______ 2. No | | | | | | | 1. *Concerns only hospitalization during the current treatment phase: For patients in continuation phase, ask only for hospitalization in this phase.* 2. *Does not include hospitalization before the current TB treatment started:*     - *For new cases, hospitalizations prior to TB treatment started should be filled in part III.*   *If answer to both question 18 and 19 are “No”, then move to question 21* | | | | | | | | | | | | | |  |
| 1. About how much money and time did you spend for each of these hospitalizations? | | | | | | | - See table below, and ask for each item. Fill one line per visit. - For all that don’t apply, mark/select NA - If there were payments for an item, but the patient cannot remember the amount, mark NR   Explanation of table headings:  Type of hospital: fill in provider type according to categories in question 6  Number of days hospitalized: includes outpatient visits as well as hospitalizations. Should be filled in chronological order  Day charges: total fees for hospital days for whole hospitalization in total. Only to be filled if not covered by the cost items below)  Consultation fee: other charges, not covered under day charge, including direct payment to health care staff  Radiography and other imaging: any imaging investigation (x-rays, CT-scan, ultrasound), TB-specific and other  Lab test fees: includes all tests, TB specific and others, including cost of transporting samples, if paid by patient  Other procedures: includes biopsy, bronchial lavage, etc. but not surgery unrelated to TB  Medicine to treat TB: fees for TB medicines only, bought inside or outside hospital  Other medicines, including nutritional supplements: any other medicine, including nutritional supplements  Out-of-pocket payments (gross): It is the sum of out-of-pocket medical and non-medical. If patient cannot remember the details of payments above, or has a hospital bill for all costs combined, ask for the total out-of-pocket payment for the hospitalization.  Out-of-pocket payment (net): sum of medical and non-medical out-of-pocket payments minus reimbursements. These net payments: should be calculated by supervisor after the interview. Not to be calculated during the interview.  Travel: out-of-pocket payment for travel to the facility (does not include income loss), for both patient and any household member.  Food: out-of-pocket payment for food bought in relation to travelling to and during the hospitalization, patient and household member.  Other, including accommodation: payments related to renting a room/bed during health care visits, and any other non-medical expenses for patient and household member.  **Health insurance reimbursement:** amount reimbursed to patient so far, does not include expected future reimbursement | | | | | | | | | | | | | | | | | | | | |  |
|  | |  |  | | | **Medical out-of-pocket payments,**  **(Total per stay)**  **(A)** | | | | | | | | | | | **Non-medical out-of-pocket payments,**  **(Total per stay)**  **(B)** | | | | | | **Out-of-pocket payments per stay (A+B)**  **(Gross)** | | **(C)** | | **Out-of-pocket payments per stay (A+B-C)**  **(Net)** | |
| Hospitalization | | Type of hospital (see list from Q17) | Number of days hospitalized | | Travel time | Day charges  (total for stay)  A1 | Consultation fee (total for stay)  A2 | | Radiography and other imaging (total for stay)  A3 | Lab tests including cost of transporting samples (total for stay)  A4 | Other procedures, including surgery, biopsy, etc.  A5 | Medicines to treat TB  (total for stay)  A6 | Other medicines, including nutritional supplements (total for stay)  A7 | | Medical payment(Total)  ΣA1-7 | | Travel  (total for stay)  B1 | Food  (total for stay)  B2 | Other (payment for linen, soap, other services & administrative)  (total for stay)  B3 | | Non-medical out-of-pocket payments (Total)  ΣB1-3 | | Total out-of-pocket payments | | Health insurance Reimbursement | |  | |
| 1^st^ | |  |  | |  |  |  | |  |  |  |  |  | |  | |  |  |  | |  | |  | |  | |  | |
| 2^nd^ | |  |  | |  |  |  | |  |  |  |  |  | |  | |  |  |  | |  | |  | |  | |  | |
| 3^rd^ | |  |  | |  |  |  | |  |  |  |  |  | |  | |  |  |  | |  | |  | |  | |  | |
| 4^th^ | |  |  | |  |  |  | |  |  |  |  |  | |  | |  |  |  | |  | |  | |  | |  | |
| 5^th^ | |  |  | |  |  |  | |  |  |  |  |  | |  | |  |  |  | |  | |  | |  | |  | |
| 6^th^ | |  |  | |  |  |  | |  |  |  |  |  | |  | |  |  |  | |  | |  | |  | |  | |
| Total hospital days  (for income loss) | | | | Σ |  |  |  |  |  |  |  |  |  |  |  |  |  |  |  |  |  |  |  |  |  |  |  |  |
| Medical out-of-pocket payments, total | | | | | | | | | | | | | | Σ A | |  |  |  |  |  |  |  |  |  |  |  |  |  |
| Non-medical out-of-pocket payment, total | | | | | | | | | | | | | | | | | | | | ΣB | |  | |  |  |  |  |  |
| Gross out-of-pocket payment | | | | | | | | | | | | | | | | | | | | | | Σ(A+B) | |  |  |  |  |  |
| Reimbursement, total | | | | | | | | | | | | | | | | | | | | | | | | ΣC | |  |  |  |
| Net out-of-pocket payment (ΣA)+( ΣB)-( ΣC), total | | | | | | | | | | | | | | | | | | | | | | | | | | Σ(A+B-C) | |  |

| ***Costs for DOT and food costs during ambulatory care*** | | |
| --- | --- | --- |
| **Question** | **Answer categories** *(check all that apply or fill answer on the answer line)* | ***Action for interviewer*** |
| 1. On a daily basis, do you currently take your medicines yourself without supervision or support (self-administered) or do you have a treatment supporter (DOT)? | 1. Self-administered  2. Home based DOT  3. Facility Based DOT | - *DOT (Directly observed treatment) visit is for the supervision of daily intake of medicines, i.e., what is done every day. These questions are not referring to less frequent trips to pick up drugs (e.g., weekly), which are explored from question 23 onwards.* - *This question concerns the treatment phase the patient is currently in* |
| 1. From Qn. 22 how many times a week? | ______________ | *The maximum will be 7 times a week* |
| 1. If you are now in the continuation phase, did you take your medicines in the intensive phase yourself without supervision or support (self-administered) or did you have a treatment supervisor or supporter (DOT)? | 1. Self-administered  2. DOT, ____ times per week | *The maximum will be 7 times a week* |
| 1. If Qn. 22 is DOT, who is the DOT provider/supporter? | 1. Health facility  2. Community health worker/volunteer  3. Workplace  4. Family member  5. Other: Specify_______________ |  |
| 1. If Qn. 22 is DOT, how long did the last DOT visit take (travel time and waiting time) total turnaround time? | _______hours |  |
| 1. What was the total cost of transport (return) incurred for the last DOT visit with your accompanying treatment supporter? | __________Tshs. |  |
| 1. How much did you spend on food and drinks for the last DOT visit (on the road, while waiting, lunch etc.), in total for you and any accompanying treatment supporter? | __________Tshs. |  |
| 1. How would you go to the health facility? | 1. Car/motorcycle 2. Public transport (bus, taxi) 3. Animal/animal cart 4. Walking 5. Bicycle 6. Other (Specify): __________ |  |
| 1. How much (if any) did you pay to the DOT provider at the last DOT visit? |  |  |
| ***Costs of picking up drugs and food costs during ambulatory care*** | | |
| **Question** | **Answer categories** *(check all that apply or fill answer on the answer line)* | ***Action for interviewer*** |
| 1. Do you or a household member pick up TB drugs (for self-administered treatment or to bring to your DOT supervisor/supporter)? | 1. Yes. 2. No | *This does not concern DOT visits, which should be recorded in questions 32-39, but should be filled if patient or other household member picks up drugs for either bringing to DOT provider or for self-administered treatment.*  *If patient is on DOT and patient or household member is* ***not*** *picking up drugs to bring to DOT provider then the answer is no.*  *If no, skip to question 34* |
| 1. If yes, how often do you or a household member pick up TB drugs in the current treatment phase? | 1. Once a day 2. Once a week 3. Once every 2 weeks 4. Once a month 5. Other specify ____________ |  |
| 1. Where do you or your household member pick up your TB drugs? | A: Public Health Facility   1. Dispensary 2. Health center 3. District Hospital 4. Regional referral Hospital 5. Zonal referral Hospital 6. National referral Hospital   B: NGO/charitable/private Facility   1. Dispensary 2. Health center 3. District Hospital 4. Regional referral Hospital 5. Zonal referral Hospital 6. Community Health Worker 7. Other specify: …………….……….. | *If the patient has visited different places, tick the most recent one.* |
| 1. How much did you pay for your anti-TB drugs during your last pick up at the facility? | __________Tshs. |  |
| 1. What accommodation cost did you and any accompanying household member have when you last picked up drugs? | __________Tshs. |  |
| 1. How long did the last visit to pick up drugs take, including travel time and waiting time (total turnaround time)? | ­­­_________ hours |  |
| 1. What was the cost of transport (return) last time you picked up drugs, including parking costs, in total for you and any accompanying household member? | __________Tshs. |  |
| 1. How much did you spend on food and drinks last time you picked up drugs (on the road, while waiting, lunch etc.), in total for you and any accompanying household member? | __________Tshs. |  |

| ***Cost during outpatient visits for medical follow-up (see the doctor or nurse, have tests)*** | | |
| --- | --- | --- |
| **Question** | **Answer categories** *(check all that apply or fill answer on the answer line)* | ***Action for interviewer*** |
| 1. How many TB-related medical follow-up visits have you had so far during this treatment phase (to see the doctor or nurse, have follow- up tests, etc.)? | ____times | *This concerns clinical check-up, follow up, and additional visits due to side effects or other TB related issues. It does not include DOT visits or visits to pick up drugs.*  *For patients in the continuation phase, ask only how many visits since the start of the intensive phase.* |
| 1. How long did the last follow-up medical outpatient visit take, including travel time and waiting time (total turnaround time)? | _____hours |  |
| 1. What was the cost of transport (return) at the last follow-up medical outpatient visit, including parking, in total for you and any accompanying household member? | __________Tshs. | *Cost related to the latest visit. If the interview takes place at the end of such a visit use the costs for the present visit* |
| 1. What accommodation cost did you incur for the last visit, in total, for you and any accompanying household member? | __________Tshs. | *Cost related to the latest visit. If the interview takes place at the end of such a visit use the costs for the present visit* |
| 1. What fees did you pay during your last follow-up medical outpatient visit for registration and or consultation? | Registration fee _____ Consultation fee_____ | *Cost related to the latest visit. If the interview takes place at the end of such a visit use the costs for the present visit* |
| 1. What fees did you pay during your last follow-up medical outpatient visit for radiography and other imaging? | __________Tshs. | *See table above for explanations* |
| 1. What fees did you pay during your last follow-up medical outpatient visit for tests, TB tests and others? | __________Tshs. | *Cost related to the latest visit. If the interview takes place at the end of such a visit use the costs for the present visit* |
| 1. What fees did you pay during your last follow-up medical outpatient visit for other procedures? | __________Tshs. | *Cost related to the latest visit. If the interview takes place at the end of such a visit use the costs for the present visit*  *See table above for explanations on other procedures* |
| 1. How much did you pay for your last follow-up medical outpatient visit for TB medicines, including prescriptions for medicines bought outside the facility? | __________Tshs. | *Cost related to the latest visit. If the interview takes place at the end of such a visit use the costs for the present visit* |
| 1. How much did you pay during your last follow-up medical outpatient visit for other medicines, including nutritional supplements? | __________Tshs. | *Cost related to the latest visit. If the interview takes place at the end of such a visit use the costs for the present visit* |
| 1. What other payment/ fees not listed in the previous questions did you pay during your last follow-up medical outpatient visit? | __________Tshs. | *Cost related to the latest visit. If the interview takes place at the end of such a visit use the costs for the present visit* |

| ***Costs for nutritional/food supplements*** | | |
| --- | --- | --- |
| **Question** | **Answer categories** *(check all that apply or fill answer on the answer line)* | ***Action for interviewer*** |
| 1. Do you buy any nutritional supplements outside your regular diet because of the TB illness, for example vitamins, meat, energy drinks, or fruits? | 1. Yes 2. No | *If no, move to question 51*  *Nutritional supplements includes micro - nutrients such as folic acid, vitamin A/ C & D, vitamin B supplements, therapeutic feeds such as F75, F100 and RUTF* |
| 1. If yes, approximately, how much did you spend on nutritional supplements in the past week? | __________Tshs. |  |
| 1. Do you buy any additional food outside of your regular diet because of TB illness, for example, meat, fruit, or energy drinks? | 1. Yes 2. No | *If no, move to question 53* |
| 1. If yes, how much do you spend on additional food in an average week approximately? | __________Tshs. |  |
|  | | |
| ***Time loss for guardians***  *Not to be filled if the patient is under 15 years – for children, all questions concerning costs, time spent, income, and income loss in sections III and IV concern cost for the guardian.*  *Note: out-of-pocket costs of transport, food, accommodation for guardian should be included in questions on Part IV (table).* | | |
| **Question** | **Answer categories** | ***Action for interviewer*** |
| 1. Did somebody in your household accompany you for your last: 2. DOT visit 3. Visit to pick up drugs (or picked up drugs for you) 4. Medical follow up visits 5. Hospitalization | 1. Yes 2. No  1. Yes 2. No  1. Yes 2. No  1. Yes 2. No | *Several responses possible*  *Time loss to be calculated with previous responses by patient* |
| 1. If yes (for any), did that person lose an income during that time? | 1. Yes 2. No | *If several responses in question 54 , ask about the latest visit when a household member accompanied* |

| ***Health Insurance*** | | |
| --- | --- | --- |
| **Question** | **Answer categories** *(check all that apply or fill answer on the answer line)* | ***Action for interviewer*** |
| 1. Do you have any of the following health insurance schemes? | 1. Family health fund 2. National health insurance fund (NHIF) 3. Social health insurance benefit (NSSF) 4. Community health fund (CHF) 5. Tiba Kwa Kadi (TIKA) 6. Private health insurance scheme, _____ (identify) 7. Other (specify) _____________ 8. Don’t know | *Private health insurance schemes such as Jubilee, Strategies, AAR, Resolution, etc*. |
| 1. How long have you been insured for? | [____\|_______] Years/months |  |
| 1. How many household members are covered by health insurance? | 1. _____ persons 2. don’t know/not sure |  |

| ***Social position*** | | |
| --- | --- | --- |
| **Question** | **Answer categories** *(circle the most appropriate or fill answer on the answer line)* | ***Action for interviewer***  *If patient is under 15 years old, these questions concern the guardian* |
| 1. What city or village do you family live in (and/or landmark)? |  | *Enter name of the place/reference points/landmarks as accurately as possible* |
| 1. Is it urban or rural area | 1. Urban 2. Rural 3. Semi urban |  |
| 1. Do you live at the same place now? | 1. Yes 2. No |  |
| 1. Have you ever attended school? | 1. Yes 2. No |  |
| 1. What is your (the patient’s) education status | 1. Pre-primary 2. Primary education 3. Post-primary training 4. Secondary “O” level 5. Post-secondary “O” level training 6. Secondary “A” level 7. University 8. Don’t know 9. Other (Specify) ____________ | *Convert to the number of ________years* |
| 1. What is the highest level you completed? | [ ] Grade |  |
| 1. What education level did the head of the household/primary income earner in the household complete? | 1. Pre-primary  2. Primary education  3. Post-primary training  4. Secondary “O” level  5. Post-secondary “O” level training  6. Secondary “A” level  7. University  8. Don’t know  9. Other (Specify) ____________ | *Convert to the number of ________years* |
| 1. What is the **main occupation** of the the head of the household/primary income earner in the household? | 1. Legislators, senior officials and managers  2. Professionals  3. Technicians and associate professionals  4. Clerks  5. Service workers and shop and market sales workers  6. Skilled agricultural and fishery workers  7. Craft and related trades workers  8. Plant and machinery operators and assemblers  9. Elementary occupations  10. Armed forces, occupations unspecified and not elsewhere classified and not economically active persons |  |
| 1. What was your primary employment, or normal work, or normal other main activity before you contracted TB? | 1. Full-time (formal/informal)  2. Part-time (formal/informal)  3. Occasional/Seasonal short-term employment  4. Unemployed – job seeking  5. Unemployment – not job seeking  6. Unable to work (sick)  7. Unable to work (disabled)  8. Student  9. Homemaker  10. Other | *This refers to the time before TB symptoms developed. Use the following options as examples to specify the responses:*  *Unemployed: student, disabled, retired*  *Self-employed: business, farming*  *Formal employment: government or private sector*  *Informal employment: housemaid, bar maid, garage mechanic* |
| 1. What is your primary employment, or normal work, or normal other main activity now? | 1. Full-time (formal/informal)  2. Part-time (formal/informal)  3. Occasional/Seasonal short-term employment  4. Unemployed – job seeking  5. Unemployment – not job seeking  6. Unable to work (sick)  7. Unable to work (disabled)  8. Student  9. Homemaker  10. Other | *This refers to the time before TB symptoms developed. Use the options provided above.* |
| ***Constructing a socio-economic status index with household asset questions*** | | |
| 1. What is your usual main source of drinking water? | 1. Bottled water 2. Tap water 3. Well 4. River/ponds 5. Other (specify): ------------ | *Other includes all sources that are not from a piped source, bottle, or well. This includes natural spring, borehole, rainwater, etc.* |
| 1. What kind of toilet facilities do you have? | 1. Flush toilet 2. Pit Latrine 3. Other (specify): ----------- |  |
| 1. Does your household have any of the following belongings? | 1. Electricity 1. Yes 2. No 2. Television 1. Yes 2. No 3. Motorcycle 1. Yes 2. No 4. Radio 1. Yes 2. No 5. Bicycle 1. Yes 2. No 6. Sewing machine 1. Yes 2. No 7. Mobile phone 1. Yes 2. No 8. Refrigerator 1. Yes 2. No 9. Car 1. Yes 2. No 10. Wrist watch 1. Yes 2. No 11. Bank account 1. Yes 2. No |  |
| 1. Is the house you are staying your own, family house or rent? | 1. Own 2. Family house 3. Rent 4. Other (specify) ____ |  |
| 1. What type of fuel does your family, which you belong to, mainly use for cooking? | 1. Electricity  3. Kerosene  4. Coal, lignite  5. Wood  6. No food cooked in the household  7. Other (Specify) ________ |  |
| 1. Is the cooking usually done in the house, in a separate building, or outdoors? | 1. In the house  2. In a separate building  3. Outdoors  4. Other (Specify) ________ |  |
| 1. What is the main material of the floor of your household? | 1. Natural floor (earth/sand/dung)  2. Rudimentary floor (wood planks)  3. Finished floor (parquet/polished wood, vinyl etc.)  4. Concrete floor  5. Other (Specify) ________________ |  |
| 1. Does your household own any livestock, herds, other farm animals, or poultry? | 1. Livestock, 2. Herds, 3. Poultry 4. Other farm animals (specify) ________ |  |
| 1. Does your household own any land (landowner)? | 1. Yes 2. No |  |
| 1. How many adult and children regularly sleep in your house? (including study participant, if variable, at time of diagnosis) | Adult # _________________  Children #_______________ |  |
| 1. How many rooms are there in the house excluding the bathroom? | # ___________________ |  |
| 1. Besides yourself, does anyone else in your household receive treatment for TB? If Yes: How many? | 1. Yes: _____ person(s) 2. No |  |

| ***Income (reported) before contracting TB*** | | | |
| --- | --- | --- | --- |
| Question | | **Answer categories** *(circle the most appropriate or fill answer on the answer line)* | ***Action for interviewer***  *If patient is under 15 years, these questions concern the guardian* |
| 1. Were you the person who earned the highest income in your household before you contracted TB? | | 1. Yes 2. No |  |
| 1. How many hours a week were you working before you contracted TB? | | __________hours | *This refers to the time before TB symptoms developed.* |
| 1. If you were in paid work, how much do you estimate your net income from labour related activities, per month was before you contracted TB? | | __________Tshs. | *In setting with an important informal sector you may not want to explicitly refer to taxes to make sure people are giving the right answer.* |
| 1. How much do you estimate the net income from labour related activities of your household was per month, before you contracted TB? | | __________Tshs. | *Refers to all persons in the household*  *In setting with an important informal sector you may not want to explicitly refer to taxes to make sure people are giving the right answer.* |
| ***Income changes and social consequences*** | | | |
| **Question** | **Answer categories** *(circle the most appropriate or fill answer on the answer line)* | | ***Action for interviewer***  *If patient is under 15 years, these questions are for the guardian* |
| 1. If you were in paid work, how much do you estimate net income from labour related activities, per month is now? | __________Tshs. | | *In setting with an important informal sector you may not want to explicitly refer to taxes to make sure people are giving the right answer.* |
| 1. How much do you estimate the net income from labour related activities of your household is per month now? | (net labour income)  __________Tshs. | | *Refers to all persons in the household* |
| 1. How many hours per week are you working now? | __________hours | |  |
| 1. Approximately how many working days of income have you lost due to your TB illness overall? | __________ days | | *Working days of income: e.g., if a patient was not able to work for 5 half days and lost income for these, the number of days lost is 0.5*5=2.5. Report for total TB episode, incl. all days before and after job loss.* |
| 1. Do you or your household receive any of the following social assistance payments? | 1. Yes  a. TASAF basic grant: _______ Tshs.  Since when: [____\|_______] month/year  1. Every week 2. Every month 3. Other (specify)___  b. TASAF conditional grant: _______ Tshs.  Since when: [____\|_______] month/year  1. Every week 2. Every month 3. Other (specify)___  c. TASAF livelihood enhancement grant: _______ Tshs.  Since when: [____\|_______] month/year  1. Every week 2. Every month 3. Other (specify)____  d. Disability benefits: _______ Tshs.  Since when: [____\|_______] month/year  1. Every week 2. Every month 3. Other (specify)____  e. Cash transfer from TB program/health facility:  _______ Tshs.  Since when: [____\|_______] month/year  1. Every week 2. Every month 3. Other (specify)____  f. Other (specify) __________________: _______ Tshs.  Since when: [____\|_______] month/year  1. Every week 2. Every month 3. Other (specify)____  2. No | | *If Yes, more than one category allowed.*  *If No, move to question 89.* |
| 1. Do you currently receive vouchers or goods in kind to cope with TB illness? | 1. Yes  a. Travel/transport voucher: ______ Tshs. per month  b. Food basket: ______ Tshs. per month  c. Nutritional support (such as vitamins, etc.): _______  Tshs. per month  d. Other:_______ Tshs. per month    2. No | | *If Yes, more than one category allowed.*  *If No, move to question 91* |
| 1. If Yes, from whom do you receive the goods | 1. Government  2. NGO  3. Employer  4. Private donation  5. Other (specify): ____________________ | | *More than one answer allowed* |
| 1. Has the TB illness affected your social or private life in any way? | 1. Yes  a. Food insecurity (*Reduction of food availability to the household)*  b. Divorce or Separated from spouse/partner  c. Loss of Job  d. Loss of income  e. Interrupted schooling  f. Social exclusion  g. Other (specify): _____________________  2. No | | *If Yes, more than one category allowed.* |

| ***Coping*** | | |
| --- | --- | --- |
| **Question** | **Answer categories** *(circle the most appropriate or fill answer on the answer line)* | ***Action for interviewer***  *If patient is under 15 years, these questions are for the guardian.* |
| 1. Did you borrow any money to cover costs due to the TB illness? | 1. Yes 2. No | *If No, go to question 96* |
| 1. If yes, how much did you borrow (in total)? | ________ Tshs. |  |
| 1. From whom did you borrow? | 1. Family  2. Neighbors/friends  3. Bank  4. VICOBA  5. Microfinance / SACCOS  6. Employer  7. Other, specify ___________________ | *More than one category allowed.* |
| 1. Are you expected to pay the loan(s) back? | 1. Yes 2. No |  |
| 1. Have you sold any of your property to finance the cost of the TB illness? | 1. Yes 2. No | *If no, skip to question 99* |
| 1. If yes, what did you sell? | 1. Land  2. Livestock  3. Vehicle/ Motorcycle/ bicycle  4. Household item  5. Farm produce  6. Jewellery  7. Other (specify):____________________ | *More than one category allowed.* |
| 1. How much money did you receive from the sale of all items of your property (in total)? |  |  |

| ***Other members of the household on treatment?*** | | |
| --- | --- | --- |
| **Question** | **Answer categories** *(circle the most appropriate or fill answer on the answer line)* | ***Action for interviewer***  *If patient is under 15 years, these questions are for the guardian.* |
| 1. Are there any members of your household currently on TB treatment? | 1. Yes ___________ (number) 2. No |  |

| *Household expenditure* | | |
| --- | --- | --- |
| **Question** | **Answer categories** *(check all that apply or fill answer on the answer line)* | *Action for interviewer* |
| In the last month, did your household spend money on the following items: | | |
| 1. Education: children school fees, books and other materials, P.T.A and other school contributions | 1. Yes ___________ (amount) 2. No |  |
| 1. Health care: clinics/HC/hospitals fees, buying drugs from private/market dispensaries, traditional/herbal treatment fees. | 1. Yes ___________ (amount) 2. No | *How much do you think you have incurred for the health care of yourself and your household members within the past one month on health other than TB?* |
| 1. Farming activities: fertilizer, insecticides, purchased of seeds, irrigation, hired labor, renting equipment, animal feeding, etc. | 1. Yes ___________ (amount) 2. No |  |
| 1. Foods: including rice, millet, maize, cassava, yam, plantain, beans groundnuts, salt, pepper, etc. | 1. Yes ___________ (amount) 2. No | *Different from additional food purchased because of TB* |
| 1. Clothing and shoes: for both adults and children | 1. Yes ___________ (amount) 2. No |  |
| 1. Utility services: water, electricity | 1. Yes ___________ (amount) 2. No |  |
| 1. Fuel: petrol, gas for cooking, kerosene, charcoal | 1. Yes ___________ (amount) 2. No |  |
| 1. Household utensils: bowls, pans, buckets, cutlery, pots and other kitchen utensils | 1. Yes ___________ (amount) 2. No |  |
| 1. Capital goods: motor vehicle, motor, bicycles, radio, buildings and building materials, grinding mills, etc. | 1. Yes ___________ (amount) 2. No |  |
| 1. Rent (only ask if person is renting house) | 1. Yes ___________ (amount) 2. No |  |
| 1. Direct taxes | 1. Yes ___________ (amount) 2. No |  |
| 1. Drinks/coolants/tobacco/funeral celebration, marriages | 1. Yes ___________ (amount) 2. No |  |
| 1. Paying debts | 1. Yes ___________ (amount) 2. No |  |
| 1. Transportation | 1. Yes ___________ (amount) 2. No |  |
| 1. Maintenance, repair, registration and insurance of motor vehicle | 1. Yes ___________ (amount) 2. No |  |
| 1. Stationery, newspaper, postage, phone cards and internet | 1. Yes ___________ (amount) 2. No |  |
| 1. Personal care items and cosmetic | 1. Yes ___________ (amount) 2. No | *Soap, shampoo, toothpaste, razor, shaving cream etc. and lipsticks, nail polish, perfume, makeup, etc.* |
| 1. Deposit into savings accounts | 1. Yes ___________ (amount) 2. No |  |
| 1. Losses from theft | 1. Yes ___________ (amount) 2. No | *Estimate value of goods* |
| 1. Gambling losses | 1. Yes ___________ (amount) 2. No |  |
| 1. Donations | 1. Yes ___________ (amount) 2. No |  |
| 1. Legal and compensation costs | 1. Yes ___________ (amount) 2. No | *Lawyer, compensation paid, court fees, etc.* |
| 1. Personal services | 1. Yes ___________ (amount) 2. No | *Haircuts, shaving, manicures, etc.* |
| 1. Others (specify) | 1. Yes ___________ (amount) 2. No |  |
| 1. Total expenditure |  | *If you they can’t give a breakdown, ask for total amount* |
| 1. What is the financial impact on your household during TB treatment, has it become: | 1 = Richer  2 = Unchanged  3 = Poorer  4 = Much poorer |  |

| **Thank you for your cooperation! Is there anything you would like to ask or say?** |
| --- |
| **Comments by Interviewer:** |
| **Date** *(dd/mm/yyyy)*: ……/……/……. |
